# Detection of circulating tumour cells with a hybrid (epithelial/mesenchymal) phenotype in patients with metastatic non-small cell lung cancer

**DOI:** 10.1038/bjc.2011.405

**Published:** 2011-10-04

**Authors:** A Lecharpentier, P Vielh, P Perez-Moreno, D Planchard, J C Soria, F Farace

**Affiliations:** 1 University of Paris-Sud, INSERM U981 ‘Identification of molecular predictors and new targets for cancer treatment’, Institut de Cancérologie Gustave Roussy, 114 rue Edouard Vaillant, 94805 Villejuif, France; 2 Translational Research Laboratory, Institut de Cancérologie Gustave Roussy, 94805 Villejuif, France; 3 Department of Medicine, Institut de Cancérologie Gustave Roussy, 94805 Villejuif, France

**Keywords:** circulating tumour cells, metastasis, metastatic non-small cell lung cancer, epithelial-mesenchymal transition, ISET

## Abstract

**Background:**

Circulating tumour cells (CTC) have a crucial role in metastasis formation and can consistently provide information on patient prognosis. Epithelial-mesenchymal transition (EMT) is considered as an essential process in the metastatic cascade, but there is currently very few data demonstrating directly the existence of the EMT process in CTCs.

**Methods:**

CTCs were enriched by blood filtration using ISET (isolation by size of epithelial tumour cells), triply labelled with fluorescent anti-vimentin, anti-pan-keratin antibodies and SYTOX orange nuclear dye, and examined by confocal microscopy in six patients with metastatic non-small cell lung cancer (NSCLC). In parallel, CTCs were morphocytologically identified by an experienced cytopathologist.

**Results:**

Isolated or clusters of dual CTCs strongly co-expressing vimentin and keratin were evidenced in all patients (range 5–88/5 ml). CTCs expressing only vimentin were detected in three patients, but were less frequent (range 3–15/5 ml). No CTC expressing only keratin was detected.

**Conclusion:**

We showed for the first time the existence of hybrid CTCs with an epithelial/mesenchymal phenotype in patients with NSCLC. Their characterisation should provide further insight on the significance of EMT in CTCs and on the mechanism of metastasis in patients with NSCLC.

Circulating tumour cells (CTCs) have an active role in the formation of metastasis and their detection can provide information on patient prognosis and treatment efficacy. Current clinical results rely exclusively on the detection and enumeration of CTCs expressing epithelial-lineage markers (i.e., the epithelial cell adhesion molecule (EpCAM and cytokeratins) in the peripheral blood (Cristofanilli *et al*, 2004; Cohen *et al*, 2008; De Bono *et al*, 2008; Mostert *et al*, 2009). Using this approach, several groups have shown an association between the CTC counts and clinical outcome of patients (Cristofanilli *et al*, 2004; Hayes *et al*, 2006; Cohen *et al*, 2008; De Bono *et al*, 2008). In most carcinomas, tumour progression implicates the loss of epithelial features and a shift towards a mesenchymal phenotype (Polyak and Weinberg, 2009; Thiery *et al*, 2009). During this process, referred to as the epithelial-mesenchymal transition (EMT) and considered as a pre-requisite to tumour infiltration and metastasis, epithelial carcinoma cells acquire a migratory phenotype and express mesenchymal genes (Polyak and Weinberg, 2009; Thiery *et al*, 2009). The phenomenon of ‘partial’ or ‘incomplete’ EMT has also been reported in which invasive and metastasising carcinoma cells may adopt a mesenchymal phenotype and express mesenchymal markers, for example, vimentin and neural cadherin while retaining epithelial charcteristics (Christiansen and Rajasekaran, 2006). However, although EMT must, by definition, have a role in the generation of at least a fraction of CTCs, there has been very few data directly demonstrating the existence of the EMT process in CTCs (Hofman *et al*, 2011; Hou *et al*, 2011). Here, we report for the first time the detection of CTCs expressing a dual epithelial and mesenchymal phenotype in the peripheral blood of patients with metastatic carcinoma.

## Patients and methods

### Blood sample collection

The present study was approved by our institutional review board and local ethics committee. Informed and written consent was obtained from all patients. Peripheral blood samples were collected from six patients with histologically or cytologically confirmed non-small cell lung cancer (NSCLC). For each patient, 10 ml of blood was collected in EDTA tubes (Terumo, Leuven, Belgium) for CTC enumeration by isolation by size of epithelial tumour cells (ISET). Peripheral blood samples were collected from six healthy volunteers for control experiments.

### Immunostaining and enumeration of CTC by ISET

Within 4 h after venopunction, blood samples were diluted 1 : 10 in an erythrocyte lysis buffer containing paraformaldehyde, filtered on the ISET device using filters with calibrated pores of 8 *μ*M (Vona *et al*, 2000; Paterlini-Brechot and Benali, 2007). Filters were air dried and conserved at −20 °C. After thawing, filters were incubated for 20 min at 98 °C in an EDTA buffer (DAKO Target retrieval buffer, pH=9.9; Dako, Glostrup, Denmark), and treated for 5 min by triton X-100 at room temperature, in a humidity chamber for cell permeabilisation. Filters were washed with TBS for 1 min and stained with markers diluted in TBS, including Alexa Fluor 488 conjugated anti-vimentin (Santa Cruz Biotechnology, Heidelberg, Germany) and Alexa Fluor 647 conjugated anti-pan-cytokeratin (Cell Signalling, Danvers, MA, USA) antibodies and SYTOX orange (Molecular Probes, Leiden, The Netherlands) overnight at 4 °C in a humidity chamber and under obscurity. SYTOX orange is a nuclear dye for which the spectra are 545 nm for excitation and 570 nm for emission. After rinsing with TBS, filters were finally mounted using Ultramount (53001; Dako). Imaging is carried out with a confocal Zeiss LSM510 Meta microscope (Carl Zeiss France, Le Pecq, France) with a × 40 or × 63 magnification and an optical slice of 1.9 *μ*m. Using the ImageJ software (NIH ImageJ, Version 1.42-q), a Gaussian blur (radius=0.10 *μ*m) was applied, and contrast was slightly enhanced in every fluorescent channels of acquisitions. CTCs were selected, digitised and validated by a senior and experienced cytopathologist (PV) as cells presenting all the following criterias: (i) nuclear size equal or larger than two pores (16 *μ*M); (ii) irregularity of the nuclear contour; (iii) presence of a visible cytoplasm; (iv) high nuclear-to-cytoplasmic ratio (greater than 0.8). A size threshold of 16 *μ*M was chosen to exclude most normal cells.

### Cell lines

The SKBR3 (breast cancer), MDA-MB-435S (breast cancer) and A549 (lung adenocarcinoma) cultured cell lines were used as controls for markers expression. All cell lines were cultured in 1640 RPMI medium supplemented 10% fetal calf serum and maintained in a humidified incubator in 5% CO_2_ at 37 °C. For control experiments, 25 cells from each cell line were precisely micro-manipulated under an inverted microscope before being spiked into blood from healthy donors. Blood samples containing spiked cell lines were then diluted using the erythrocyte lysis buffer, filtered and stained as described for patient samples.

### Immunohistochemical staining of tumours

Immunohistochemical stainings were performed on sections of formalin-fixed paraffin-embedded samples, using cytokeratin 7 (Dako) and vimentin (Santa Cruz Biotechnology) according to standard procedures.

## Results

**Figure 1:**
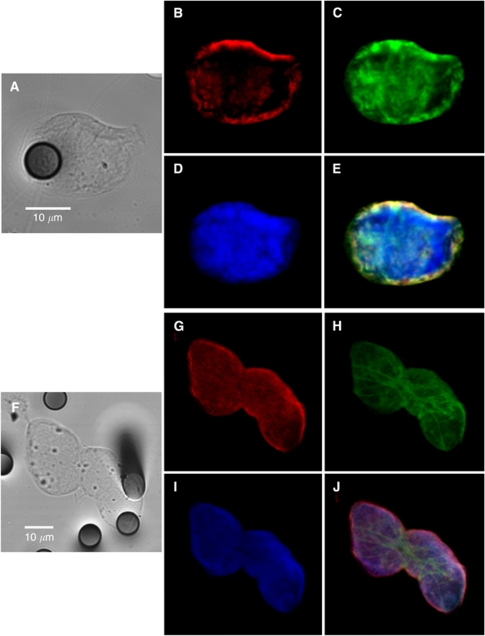
Confocal microscopic analysis of CTCs expressing a hybrid phenotype. Examples of an isolated CTC (**A**–**E**) and of a cluster of CTCs (**F**–**J**) from two patients with metastatic NSCLC. Tumour morphology is visualised by equatorial *Z*-plane bright field image (**A** and **F**). Keratins (**B** and **G**), vimentin (**C** and **H**) and equatorial *Z*-plane nucleus (**D**–**I**) images are merged on **E** and **J**. Optical slice thickness allows the detection of both nuclear and cytoplasmic signals. During analysis using ImageJ, colours were assigned arbitrarily, that is, green, red and blue colours for AF488, AF647 and SYTOX orange, respectively. The 8-*μ*m wide pores are visible on bright field images.

**Figure 2:**
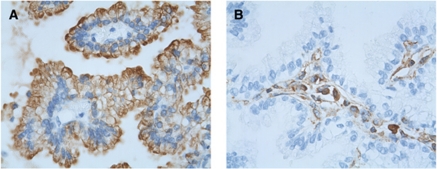
Primary lung adenocarcinoma (patient 6). (**A**) Cytokeratin 7 immunostaining showing positive tumour cells surrounding negative conjonctive tissue; (**B**) Vimentin immunostaining performed on a serial section of the same tumour, showing that malignant cells are negative, whereas conjonctive tissue is stained.

Blood samples from six patients with metastatic NSCLC were processed on the ISET device, which allows CTC enrichment by blood filtration through filters with calibrated pores (Vona *et al*, 2000; Paterlini-Brechot and Benali, 2007). Triple fluorescent labelling with anti-vimentin, anti-pan-keratin antibodies and SYTOX orange for nuclear staining was performed to detect CTCs using confocal microscopy. Control experiments were performed in parallel using 25 cells of SKBR3 (pan-keratin positive), MDA-MB-435S (vimentin positive) and A549 (pan-keratin and vimentin positive) cell lines spiked in blood from healthy donors (Table 1). Blood samples from six healthy donors were also processed by ISET and labelled identically. Isolated and/or clusters of immunostained CTCs were morphocytologically identified by an experienced cytopathologist (PV) using bright field images (Figure 1A and F). A strong co-expression of vimentin and pan-keratin antigens (Figure 1B–E and G–J) was observed in almost all CTCs from the six NSCLC patients. Counts of dual CTCs co-expressing pan-keratin and vimentin, of only vimentin positive and only pan-keratin positive CTCs in the six NSCLC patients are presented in Table 1. Dual CTCs expressing both pan-keratin and vimentin were mainly present in clusters and ranged from 5 to 88 cells per 5 ml. CTCs expressing only pan-keratin antigens were not detected in any of the patients, whereas CTCs expressing only vimentin were more rare and detected in three patients (range 3–15/5 ml). The phenotype detected here in CTCs therefore strongly differed from that detected in primary tumours in NSCLC patients, which are positive for pan-keratin antigens and negative for vimentin. As an example, Figure 2A and B shows histological staining from a patient with lung primary adenocarcinoma (patient 6) presenting a keratin-positive and vimentin-negative phenotype. No CTCs were detected in the blood of healthy donors.

## Conclusion

Our exploratory study demonstrates for the first time that the majority of isolated or clusters of CTCs in patients with advanced metastatatic NSCLC harbour a dual epithelial–mesenchymal phenotype, suggesting that EMT is a relevant process for invasion and metastatasis in these patients. CTCs expressing only vimentin (or co-expressing also keratin antigens at a very low yet undetectable level) were also observed. These data underscore the reality of EMT in CTCs and provide experimental evidence to the paradigm of a phenotypical continuum between epithelial and mesenchymal states in CTCs (Mego *et al*, 2010). Low counts of CTCs have been reported in NSCLC patients by methods using EpCAM antigen expression-based enrichment (Allard *et al*, 2004). The partial loss of epithelial markers could therefore lead to an underestimation of CTCs with hybrid phenotype, as previously shown using breast cancer cell lines (Sieuwerts *et al*, 2009). EMT phenotype is associated with stem cell properties and resistance to anticancer treatment (Polyak and Weinberg, 2009; Thiery *et al*, 2009). A better phenotypical and molecular characterisation of CTCs with hybrid phenotype should provide further insight on the significance of EMT in CTCs and on the molecular mechanism of metastasis in patients with NSCLC.

## Figures and Tables

**Table 1:** Counts of CTC subpopulations according to keratins and vimentin expression in NSCLC patient

				**Keratins+ vimentin+ SYTOX+ CTC counts (per 5 ml)**					
	**Primary tumour**	**Time between diagnosis and CTC analysis (months)**	**Total CTC counts (per 5 ml)**	**Isolated CTC**	**CTC in clusters^a^**	**Keratins+ SYTOX+ CTC counts (per 5 ml)**	**Vimentin+ SYTOX+ CTC counts (per 5 ml)**	**SYTOX+ excluded events^b^ (per 5 ml)**	**Unassigned SYTOX+ cells^c^ (per 5 ml)**
*Patients*									
Patient 1	Adenocarcinoma	32	40	8	32 (10)	0	0	0	11 990
Patient 2	Large cell carcinoma	50	95	3	85 (20)	0	7	38	3120
Patient 3	Adenocarcinoma	30	40	20	5 (3)	0	15	25	7730
Patient 4	Adenocarcinoma	8.5	8	3	5 (3)	0	0	70	6325
Patient 5	Small cell carcinoma	18	70	18	52 (18)	0	0	35	4780
Patient 6	Adenocarcinoma with BAC features	7.5	8	5	0	0	3	0	7340
									
*Control cell lines*									
SKBR3	—	—	24	0	0	24	0	0	343
A549	—	—	22	22	0	0	0	0	877
MDA-MB-435S	—	—	21	0	0	0	21	0	863
									
*Donors*									
D1	—	—	0	0	0	0	0	0	3980
D2	—	—	0	0	0	0	0	0	765
D3	—	—	0	0	0	0	0	0	5770
D4	—	—	0	0	0	0	0	0	9615
D5	—	—	0	0	0	0	0	0	5010
D6	—	—	0	0	0	0	0	0	3295

Abbreviations: BAC=bronchioloalveolar carcinoma; CTC=circulating tumour cells; NSCLC=non-small cell lung cancer.

a The number of clusters is indicated in brackets.

b SYTOX+ excluded events were large intact nucleus without cytoplasm, which were keratin-negative and vimentin-negative.

c Unassigned SYTOX+ cells were intact small cells with a nucleus and cytoplasm, which were most likely haematopoietic cells. In almost all the case, unassigned SYTOX+ cells were vimentin+. Unassigned SYTOX cells were counted in one spot of the filter corresponding to 1 ml blood. Values obtained for 5 ml were calculated using values obtained in 1 ml.
